# Methyl 3-(4-{6-methyl-4-[3-(trifluoro­meth­yl)phen­yl]pyridazin-3-yl­oxy}phen­yl)propanoate

**DOI:** 10.1107/S1600536808013342

**Published:** 2008-05-10

**Authors:** Yu-Hong Xiao, You-Quan Zhu, Xiao-Mao Zou, Fang-Zhong Hu, Hua-Zheng Yang

**Affiliations:** a State Key Laboratory and Institute of Elemento-Organic Chemistry, Nankai University, Tianjin 300071, People’s Republic of China

## Abstract

In the title compound, C_22_H_19_F_3_N_2_O_3_, the benzene rings of the trifluoro­methyl­phenyl and benzoyl­phenyl groups form dihedral angles of 41.89 (10) and 67.44 (10)°, respectively, with the pyridazine ring. The methyl­propanoate group is nearly coplanar with the attached benzene ring [dihedral angle = 3.9 (2)°]. The trifluoro­methyl group is disordered over two positions; the site-occupancy factors are *ca* 0.64 and 0.36. In the crystal structure, inversion-related mol­ecules are linked through C—H⋯O hydrogen bonds and C—H⋯π inter­actions.

## Related literature

For the biological activities of pyridazine derivatives, see: Heinisch & Kopelent (1992 ▶); Kolar & Tisler (1999 ▶).
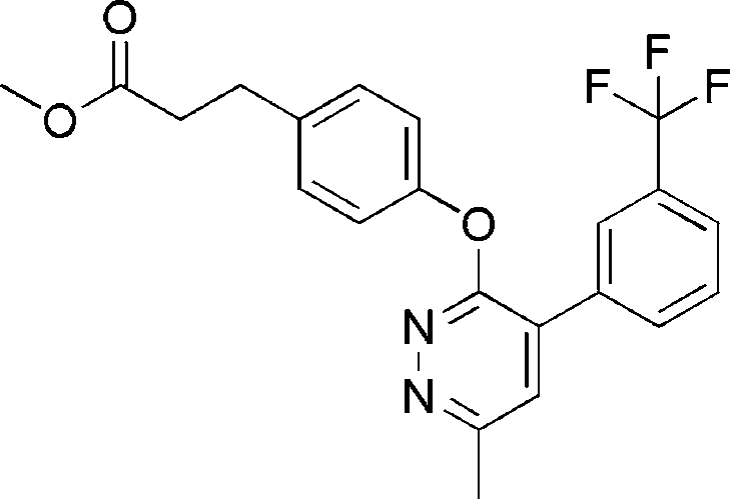


## Experimental

### 

#### Crystal data

C_22_H_19_F_3_N_2_O_3_
*M* *_r_* = 416.39
Triclinic, 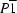

*a* = 9.4916 (19) Å
*b* = 10.232 (2) Å
*c* = 11.022 (2) Å
α = 81.70 (3)°
β = 65.12 (3)°
γ = 78.92 (3)°
*V* = 950.6 (4) Å^3^
*Z* = 2
Mo *K*α radiation
μ = 0.12 mm^−1^
*T* = 113 (2) K
0.20 × 0.16 × 0.06 mm

#### Data collection

Rigaku Saturn CCD area-detector diffractometer
Absorption correction: multi-scan (*CrystalClear*; Rigaku/MSC, 2005 ▶) *T*_min_ = 0.977, *T*_max_ = 0.993
5529 measured reflections
3319 independent reflections
1862 reflections with *I* > 2σ(*I*)
*R*_int_ = 0.054

#### Refinement

*R*[*F* ^2^ > 2σ(*F* ^2^)] = 0.055
*wR*(*F* ^2^) = 0.147
*S* = 1.06
3319 reflections
301 parameters
72 restraints
H-atom parameters constrained
Δρ_max_ = 0.40 e Å^−3^
Δρ_min_ = −0.39 e Å^−3^

### 

Data collection: *CrystalClear* (Rigaku/MSC, 2005 ▶); cell refinement: *CrystalClear*; data reduction: *CrystalClear*; program(s) used to solve structure: *SHELXS97* (Sheldrick, 2008 ▶); program(s) used to refine structure: *SHELXL97* (Sheldrick, 2008 ▶); molecular graphics: *SHELXTL* (Sheldrick, 2008 ▶); software used to prepare material for publication: *SHELXL97*.

## Supplementary Material

Crystal structure: contains datablocks global, I. DOI: 10.1107/S1600536808013342/ci2581sup1.cif

Structure factors: contains datablocks I. DOI: 10.1107/S1600536808013342/ci2581Isup2.hkl

Additional supplementary materials:  crystallographic information; 3D view; checkCIF report

## Figures and Tables

**Table 1:** Hydrogen-bond geometry (Å, °)

*D*—H⋯*A*	*D*—H	H⋯*A*	*D*⋯*A*	*D*—H⋯*A*
C11—H11*B*⋯O2^i^	0.96	2.57	3.467 (4)	156
C22—H22*C*⋯*Cg*1^ii^	0.96	2.73	3.486 (4)	136

Symmetry codes: (i) 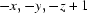; (ii) 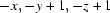. *Cg*1 is the centroid of the C2–C7 ring.

**Fig. 1.**
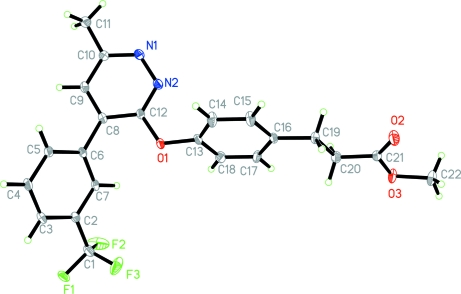
The molecular structure of the title compound, showing 30% probability displacement ellipsoids. Only one disorder component is shown.

## supplementary crystallographic information

### Comment

Many pyridazine derivatives have been found to exhibit biological activities such as insecticidal, fungicidal, herbicidal, plant-growth regulatory activity, *etc* (Heinisch & Kopelent, 1992). For example, pyridate, credazine and maleic hydrazide (Kolar & Tisler, 1999) have been commercialized as herbicides. In order to discover new biologically active pyridazine compounds, the title compound was synthesized and its structure is reported here.

In the title molecule (Fig. 1), the C2—C7 and C13—C18 benzene rings form dihedral angles of 41.89 (10)° and 67.44 (10)°, respectively, with the central pyridazine ring (C2—C4/C12/N1/N2). The methylpropanoate group is nearly coplanar with the C13—C18 benzene ring (dihedral angle 3.9 (2)°).

The crystal packing is stabilized by C—H···O hydrogen bonds and C—H···π interactions (Table 1) involving the C2—C7 ring (centroid *Cg*1).

### Experimental

A mixture of methyl 3-(4-hydroxyphenyl)propanoate (2.3 mmol), 3-chloro-4- (3-(trifluoromethyl)phenyl)-6-methylpyridazine (2.3 mmol) and K_2_CO_3_ (5 mmol) was stirred at 313 K for 2 h. The crude product was recrystallized from ethanol and single crystals of the title compound suitable for X-ray analysis were grown from ethyl acetate-petroleum ether (3:1 *v*/*v*) at room temperature.

### Refinement

The trifluoromethyl group is disordered over two orienatations (C1/F1/F2/F3 and C1/F1'/F2'/F3') with refined occupancies of 0.636 (12) and 0.364 (12). All C—F distances were restrained to 1.34 (1) Å and the U^ij^ components of disordered F atoms were restrained to be approximately isotropic. All H atoms were positioned geometrically (C—H = 0.93–0.97 Å) and included in the final cycles of refinement using a riding model, with *U*_iso_(H) = 1.2*U*_eq_(C) or 1.5*U*_eq_(methyl C).

### Crystal data

**Table:**

C_22_H_19_F_3_N_2_O_3_	*Z* = 2
*M**_r_* = 416.39	*F*_000_ = 432
Triclinic, *P*1	*D*_x_ = 1.455 Mg m^−^^3^
Hall symbol: -P 1	Mo *K*α radiation λ = 0.71073 Å
*a* = 9.4916 (19) Å	Cell parameters from 2351 reflections
*b* = 10.232 (2) Å	θ = 2.0–27.9º
*c* = 11.022 (2) Å	µ = 0.12 mm^−^^1^
α = 81.70 (3)º	*T* = 113 (2) K
β = 65.12 (3)º	Plate, colourless
γ = 78.92 (3)º	0.20 × 0.16 × 0.06 mm
*V* = 950.6 (4) Å^3^	

### Data collection

**Table:**

Rigaku Saturn CCD area-detector diffractometer	3319 independent reflections
Radiation source: rotating anode	1862 reflections with *I* > 2σ(*I*)
Monochromator: confocal	*R*_int_ = 0.054
Detector resolution: 7.31 pixels mm^-1^	θ_max_ = 25.0º
*T* = 113(2) K	θ_min_ = 2.0º
ω and φ scans	*h* = −10→11
Absorption correction: multi-scan(CrystalClear; Rigaku/MSC, 2005)	*k* = −12→11
*T*_min_ = 0.977, *T*_max_ = 0.993	*l* = −13→11
5529 measured reflections	

### Refinement

**Table:**

Refinement on *F*^2^	Secondary atom site location: difference Fourier map
Least-squares matrix: full	Hydrogen site location: inferred from neighbouring sites
*R*[*F*^2^ > 2σ(*F*^2^)] = 0.055	H-atom parameters constrained
*wR*(*F*^2^) = 0.147	*w* = 1/[σ^2^(*F*_o_^2^) + (0.0675*P*)^2^] where *P* = (*F*_o_^2^ + 2*F*_c_^2^)/3
*S* = 1.06	(Δ/σ)_max_ = 0.003
3319 reflections	Δρ_max_ = 0.40 e Å^−^^3^
301 parameters	Δρ_min_ = −0.39 e Å^−^^3^
72 restraints	Extinction correction: none
Primary atom site location: structure-invariant direct methods	

### Special details

**Table:**

Geometry. All e.s.d.'s (except the e.s.d. in the dihedral angle between two l.s. planes) are estimated using the full covariance matrix. The cell e.s.d.'s are taken into account individually in the estimation of e.s.d.'s in distances, angles and torsion angles; correlations between e.s.d.'s in cell parameters are only used when they are defined by crystal symmetry. An approximate (isotropic) treatment of cell e.s.d.'s is used for estimating e.s.d.'s involving l.s. planes.
Refinement. Refinement of *F*^2^ against ALL reflections. The weighted *R*-factor *wR* and goodness of fit *S* are based on *F*^2^, conventional *R*-factors *R* are based on *F*, with *F* set to zero for negative *F*^2^. The threshold expression of *F*^2^ > σ(*F*^2^) is used only for calculating *R*-factors(gt) *etc*. and is not relevant to the choice of reflections for refinement. *R*-factors based on *F*^2^ are statistically about twice as large as those based on *F*, and *R*- factors based on ALL data will be even larger.

### Fractional atomic coordinates and isotropic or equivalent isotropic displacement parameters (Å^2^)

**Table:**

	*x*	*y*	*z*	*U*_iso_*/*U*_eq_	Occ. (<1)
F1	1.0340 (5)	0.3434 (7)	0.0132 (7)	0.0359 (15)	0.636 (12)
F2	0.8747 (11)	0.3445 (7)	−0.0736 (7)	0.072 (2)	0.636 (12)
F3	0.7991 (6)	0.4176 (6)	0.1223 (8)	0.079 (3)	0.636 (12)
F1'	1.0297 (11)	0.3418 (14)	−0.0418 (14)	0.071 (4)	0.364 (12)
F2'	0.7900 (12)	0.3936 (8)	−0.0042 (15)	0.055 (3)	0.364 (12)
F3'	0.8553 (19)	0.4059 (12)	0.1421 (11)	0.076 (4)	0.364 (12)
O1	0.39298 (18)	0.1498 (2)	0.2418 (2)	0.0286 (6)	
O2	−0.5057 (2)	0.5985 (2)	0.6250 (2)	0.0409 (7)	
O3	−0.4466 (2)	0.7531 (2)	0.4543 (2)	0.0330 (6)	
N1	0.3114 (2)	−0.1545 (3)	0.1899 (3)	0.0246 (7)	
N2	0.2934 (2)	−0.0267 (3)	0.2179 (3)	0.0251 (7)	
C1	0.8892 (3)	0.3235 (3)	0.0444 (3)	0.0327 (9)	
C2	0.8618 (3)	0.1868 (3)	0.1025 (3)	0.0228 (8)	
C3	0.9650 (3)	0.1072 (3)	0.1502 (3)	0.0258 (8)	
H3	1.0518	0.1399	0.1464	0.031*	
C4	0.9393 (3)	−0.0202 (3)	0.2033 (3)	0.0266 (8)	
H4	1.0083	−0.0738	0.2361	0.032*	
C5	0.8108 (3)	−0.0688 (3)	0.2079 (3)	0.0241 (8)	
H5	0.7947	−0.1555	0.2430	0.029*	
C6	0.7056 (2)	0.0103 (3)	0.1608 (3)	0.0194 (7)	
C7	0.7313 (3)	0.1389 (3)	0.1080 (3)	0.0205 (7)	
H7	0.6616	0.1931	0.0763	0.025*	
C8	0.5697 (3)	−0.0462 (3)	0.1662 (3)	0.0191 (7)	
C9	0.5840 (3)	−0.1748 (3)	0.1379 (3)	0.0227 (7)	
H9	0.6818	−0.2278	0.1092	0.027*	
C10	0.4511 (3)	−0.2271 (3)	0.1519 (3)	0.0216 (7)	
C11	0.4609 (3)	−0.3680 (3)	0.1257 (3)	0.0319 (9)	
H11A	0.3575	−0.3868	0.1466	0.048*	
H11B	0.5055	−0.4269	0.1806	0.048*	
H11C	0.5259	−0.3811	0.0329	0.048*	
C12	0.4159 (3)	0.0228 (3)	0.2087 (3)	0.0202 (7)	
C13	0.2387 (3)	0.2146 (3)	0.3058 (3)	0.0260 (8)	
C14	0.1408 (3)	0.1712 (3)	0.4317 (3)	0.0353 (10)	
H14	0.1721	0.0931	0.4743	0.042*	
C15	−0.0044 (3)	0.2448 (3)	0.4941 (3)	0.0340 (9)	
H15	−0.0710	0.2150	0.5792	0.041*	
C16	−0.0543 (3)	0.3619 (3)	0.4340 (3)	0.0215 (7)	
C17	0.0473 (3)	0.4015 (3)	0.3070 (3)	0.0311 (9)	
H17	0.0168	0.4794	0.2636	0.037*	
C18	0.1928 (3)	0.3285 (3)	0.2428 (3)	0.0324 (9)	
H18	0.2593	0.3568	0.1570	0.039*	
C19	−0.2110 (3)	0.4412 (3)	0.5096 (3)	0.0250 (8)	
H19A	−0.2905	0.3833	0.5370	0.030*	
H19B	−0.2102	0.4673	0.5905	0.030*	
C20	−0.2594 (3)	0.5645 (3)	0.4362 (3)	0.0287 (8)	
H20A	−0.1815	0.6240	0.4094	0.034*	
H20B	−0.2615	0.5396	0.3554	0.034*	
C21	−0.4164 (3)	0.6373 (3)	0.5179 (3)	0.0246 (8)	
C22	−0.5929 (3)	0.8355 (3)	0.5257 (4)	0.0392 (9)	
H22A	−0.6786	0.7956	0.5298	0.059*	
H22B	−0.5942	0.9226	0.4798	0.059*	
H22C	−0.6034	0.8431	0.6150	0.059*	

### Atomic displacement parameters (Å^2^)

**Table:**

	*U*^11^	*U*^22^	*U*^33^	*U*^12^	*U*^13^	*U*^23^
F1	0.0233 (17)	0.036 (2)	0.049 (3)	−0.0158 (15)	−0.0157 (19)	0.011 (3)
F2	0.123 (5)	0.063 (4)	0.071 (4)	−0.058 (3)	−0.075 (4)	0.042 (3)
F3	0.041 (3)	0.028 (3)	0.102 (5)	0.003 (2)	0.027 (3)	0.006 (3)
F1'	0.052 (4)	0.035 (4)	0.069 (7)	−0.004 (3)	0.025 (4)	0.012 (5)
F2'	0.057 (4)	0.035 (4)	0.093 (7)	−0.009 (3)	−0.056 (4)	0.019 (4)
F3'	0.129 (8)	0.040 (5)	0.051 (5)	−0.008 (6)	−0.026 (6)	−0.014 (4)
O1	0.0136 (9)	0.0279 (13)	0.0422 (15)	0.0010 (8)	−0.0076 (9)	−0.0135 (11)
O2	0.0284 (11)	0.0378 (15)	0.0377 (16)	0.0032 (10)	−0.0012 (10)	0.0053 (12)
O3	0.0283 (10)	0.0297 (13)	0.0279 (14)	0.0109 (9)	−0.0045 (9)	−0.0039 (11)
N1	0.0212 (11)	0.0294 (15)	0.0253 (16)	−0.0046 (11)	−0.0108 (10)	−0.0031 (12)
N2	0.0198 (11)	0.0284 (16)	0.0286 (16)	−0.0037 (10)	−0.0104 (11)	−0.0046 (12)
C1	0.0232 (15)	0.038 (2)	0.037 (2)	−0.0089 (15)	−0.0131 (15)	0.0076 (19)
C2	0.0160 (12)	0.0280 (18)	0.0207 (18)	−0.0029 (11)	−0.0039 (11)	−0.0019 (14)
C3	0.0154 (12)	0.0360 (19)	0.0261 (19)	−0.0052 (12)	−0.0083 (12)	−0.0008 (15)
C4	0.0179 (13)	0.0323 (19)	0.030 (2)	−0.0012 (12)	−0.0133 (12)	0.0042 (15)
C5	0.0179 (12)	0.0241 (17)	0.0269 (19)	−0.0018 (11)	−0.0070 (12)	0.0003 (14)
C6	0.0124 (12)	0.0271 (17)	0.0154 (16)	−0.0008 (11)	−0.0022 (11)	−0.0053 (13)
C7	0.0146 (12)	0.0280 (18)	0.0161 (17)	0.0013 (11)	−0.0050 (11)	−0.0031 (13)
C8	0.0159 (12)	0.0283 (18)	0.0124 (17)	−0.0022 (12)	−0.0054 (11)	−0.0016 (14)
C9	0.0180 (12)	0.0278 (18)	0.0191 (18)	0.0007 (12)	−0.0059 (12)	−0.0024 (14)
C10	0.0226 (13)	0.0246 (17)	0.0169 (17)	−0.0042 (12)	−0.0069 (12)	−0.0013 (13)
C11	0.0278 (14)	0.0296 (19)	0.035 (2)	−0.0048 (13)	−0.0091 (14)	−0.0047 (16)
C12	0.0156 (12)	0.0246 (17)	0.0194 (18)	0.0002 (12)	−0.0059 (11)	−0.0059 (14)
C13	0.0140 (12)	0.0303 (18)	0.034 (2)	−0.0019 (12)	−0.0080 (12)	−0.0097 (15)
C14	0.0269 (15)	0.040 (2)	0.033 (2)	0.0083 (14)	−0.0122 (14)	0.0010 (17)
C15	0.0232 (14)	0.041 (2)	0.026 (2)	0.0040 (13)	−0.0049 (13)	0.0035 (16)
C16	0.0151 (12)	0.0253 (17)	0.0235 (18)	−0.0003 (11)	−0.0064 (12)	−0.0079 (14)
C17	0.0251 (14)	0.0235 (18)	0.031 (2)	0.0039 (13)	−0.0021 (13)	0.0000 (15)
C18	0.0244 (14)	0.0294 (19)	0.031 (2)	−0.0042 (13)	0.0008 (13)	0.0002 (16)
C19	0.0210 (13)	0.0270 (18)	0.0226 (18)	0.0019 (12)	−0.0057 (12)	−0.0051 (14)
C20	0.0255 (14)	0.0269 (18)	0.029 (2)	−0.0005 (12)	−0.0073 (13)	−0.0027 (15)
C21	0.0216 (13)	0.0245 (18)	0.027 (2)	−0.0018 (12)	−0.0091 (13)	−0.0046 (15)
C22	0.0351 (16)	0.035 (2)	0.039 (2)	0.0139 (14)	−0.0126 (15)	−0.0098 (17)

### Geometric parameters (Å, °)

**Table:**

F1—C1	1.319 (5)	C8—C12	1.405 (3)
F2—C1	1.347 (5)	C9—C10	1.405 (4)
F3—C1	1.304 (7)	C9—H9	0.93
F1'—C1	1.302 (8)	C10—C11	1.489 (4)
F2'—C1	1.320 (6)	C11—H11A	0.96
F3'—C1	1.357 (9)	C11—H11B	0.96
O1—C12	1.353 (4)	C11—H11C	0.96
O1—C13	1.405 (3)	C13—C18	1.363 (4)
O2—C21	1.192 (3)	C13—C14	1.372 (4)
O3—C21	1.333 (3)	C14—C15	1.375 (4)
O3—C22	1.442 (3)	C14—H14	0.93
N1—C10	1.316 (3)	C15—C16	1.384 (4)
N1—N2	1.348 (4)	C15—H15	0.93
N2—C12	1.317 (3)	C16—C17	1.380 (4)
C1—C2	1.475 (4)	C16—C19	1.502 (4)
C2—C3	1.379 (3)	C17—C18	1.379 (4)
C2—C7	1.393 (4)	C17—H17	0.93
C3—C4	1.372 (4)	C18—H18	0.93
C3—H3	0.93	C19—C20	1.501 (4)
C4—C5	1.382 (4)	C19—H19A	0.97
C4—H4	0.93	C19—H19B	0.97
C5—C6	1.388 (3)	C20—C21	1.491 (4)
C5—H5	0.93	C20—H20A	0.97
C6—C7	1.382 (4)	C20—H20B	0.97
C6—C8	1.488 (4)	C22—H22A	0.96
C7—H7	0.93	C22—H22B	0.96
C8—C9	1.364 (4)	C22—H22C	0.96
			
C12—O1—C13	119.0 (2)	C10—C11—H11A	109.5
C21—O3—C22	116.2 (2)	C10—C11—H11B	109.5
C10—N1—N2	119.7 (2)	H11A—C11—H11B	109.5
C12—N2—N1	119.3 (2)	C10—C11—H11C	109.5
F1'—C1—F3	119.9 (8)	H11A—C11—H11C	109.5
F3—C1—F1	105.4 (5)	H11B—C11—H11C	109.5
F1'—C1—F2'	106.9 (7)	N2—C12—O1	117.9 (2)
F3—C1—F2'	69.2 (6)	N2—C12—C8	124.8 (3)
F1—C1—F2'	126.6 (5)	O1—C12—C8	117.2 (2)
F1'—C1—F2	76.7 (7)	C18—C13—C14	120.7 (3)
F3—C1—F2	109.6 (4)	C18—C13—O1	117.6 (2)
F1—C1—F2	103.9 (4)	C14—C13—O1	121.6 (2)
F1'—C1—F3'	104.6 (9)	C13—C14—C15	119.1 (3)
F1—C1—F3'	82.7 (7)	C13—C14—H14	120.5
F2'—C1—F3'	95.6 (7)	C15—C14—H14	120.5
F2—C1—F3'	132.0 (6)	C14—C15—C16	122.0 (3)
F1'—C1—C2	118.4 (6)	C14—C15—H15	119.0
F3—C1—C2	114.6 (4)	C16—C15—H15	119.0
F1—C1—C2	112.6 (4)	C17—C16—C15	117.1 (3)
F2'—C1—C2	117.7 (4)	C17—C16—C19	123.3 (2)
F2—C1—C2	110.1 (4)	C15—C16—C19	119.5 (2)
F3'—C1—C2	110.7 (6)	C18—C17—C16	121.6 (3)
C3—C2—C7	120.5 (3)	C18—C17—H17	119.2
C3—C2—C1	120.2 (2)	C16—C17—H17	119.2
C7—C2—C1	119.4 (2)	C13—C18—C17	119.6 (3)
C4—C3—C2	119.8 (2)	C13—C18—H18	120.2
C4—C3—H3	120.1	C17—C18—H18	120.2
C2—C3—H3	120.1	C20—C19—C16	116.4 (2)
C3—C4—C5	120.0 (2)	C20—C19—H19A	108.2
C3—C4—H4	120.0	C16—C19—H19A	108.2
C5—C4—H4	120.0	C20—C19—H19B	108.2
C4—C5—C6	120.8 (3)	C16—C19—H19B	108.2
C4—C5—H5	119.6	H19A—C19—H19B	107.3
C6—C5—H5	119.6	C21—C20—C19	113.1 (2)
C7—C6—C5	119.1 (2)	C21—C20—H20A	109.0
C7—C6—C8	121.7 (2)	C19—C20—H20A	109.0
C5—C6—C8	119.2 (2)	C21—C20—H20B	109.0
C6—C7—C2	119.8 (2)	C19—C20—H20B	109.0
C6—C7—H7	120.1	H20A—C20—H20B	107.8
C2—C7—H7	120.1	O2—C21—O3	123.5 (3)
C9—C8—C12	114.3 (2)	O2—C21—C20	125.7 (2)
C9—C8—C6	121.7 (2)	O3—C21—C20	110.9 (2)
C12—C8—C6	123.9 (3)	O3—C22—H22A	109.5
C8—C9—C10	120.0 (2)	O3—C22—H22B	109.5
C8—C9—H9	120.0	H22A—C22—H22B	109.5
C10—C9—H9	120.0	O3—C22—H22C	109.5
N1—C10—C9	121.7 (3)	H22A—C22—H22C	109.5
N1—C10—C11	116.6 (2)	H22B—C22—H22C	109.5
C9—C10—C11	121.7 (2)		
			
C10—N1—N2—C12	−1.1 (4)	N2—N1—C10—C11	178.8 (3)
F1'—C1—C2—C3	46.6 (11)	C8—C9—C10—N1	1.2 (4)
F3—C1—C2—C3	−104.0 (6)	C8—C9—C10—C11	−178.3 (3)
F1—C1—C2—C3	16.5 (6)	N1—N2—C12—O1	−177.8 (2)
F2'—C1—C2—C3	177.6 (8)	N1—N2—C12—C8	2.6 (4)
F2—C1—C2—C3	131.9 (6)	C13—O1—C12—N2	11.0 (4)
F3'—C1—C2—C3	−74.0 (8)	C13—O1—C12—C8	−169.4 (2)
F1'—C1—C2—C7	−133.2 (10)	C9—C8—C12—N2	−2.1 (4)
F3—C1—C2—C7	76.3 (6)	C6—C8—C12—N2	−178.0 (3)
F1—C1—C2—C7	−163.2 (5)	C9—C8—C12—O1	178.3 (3)
F2'—C1—C2—C7	−2.2 (9)	C6—C8—C12—O1	2.5 (4)
F2—C1—C2—C7	−47.8 (6)	C12—O1—C13—C18	−119.9 (3)
F3'—C1—C2—C7	106.2 (8)	C12—O1—C13—C14	64.9 (4)
C7—C2—C3—C4	0.2 (5)	C18—C13—C14—C15	−0.4 (5)
C1—C2—C3—C4	−179.6 (3)	O1—C13—C14—C15	174.6 (3)
C2—C3—C4—C5	0.5 (5)	C13—C14—C15—C16	−0.4 (5)
C3—C4—C5—C6	−0.8 (5)	C14—C15—C16—C17	0.9 (5)
C4—C5—C6—C7	0.5 (5)	C14—C15—C16—C19	−177.2 (3)
C4—C5—C6—C8	179.4 (3)	C15—C16—C17—C18	−0.5 (5)
C5—C6—C7—C2	0.2 (5)	C19—C16—C17—C18	177.4 (3)
C8—C6—C7—C2	−178.7 (3)	C14—C13—C18—C17	0.7 (5)
C3—C2—C7—C6	−0.5 (5)	O1—C13—C18—C17	−174.5 (3)
C1—C2—C7—C6	179.3 (3)	C16—C17—C18—C13	−0.3 (5)
C7—C6—C8—C9	139.4 (3)	C17—C16—C19—C20	4.1 (5)
C5—C6—C8—C9	−39.5 (4)	C15—C16—C19—C20	−178.0 (3)
C7—C6—C8—C12	−45.0 (4)	C16—C19—C20—C21	179.8 (3)
C5—C6—C8—C12	136.1 (3)	C22—O3—C21—O2	3.2 (5)
C12—C8—C9—C10	0.1 (4)	C22—O3—C21—C20	−177.6 (3)
C6—C8—C9—C10	176.1 (2)	C19—C20—C21—O2	−7.7 (5)
N2—N1—C10—C9	−0.8 (4)	C19—C20—C21—O3	173.1 (3)

### Hydrogen-bond geometry (Å, °)

**Table:**

*D*—H···*A*	*D*—H	H···*A*	*D*···*A*	*D*—H···*A*
C11—H11B···O2^i^	0.96	2.57	3.467 (4)	156
C22—H22C···Cg1^ii^	0.96	2.73	3.486 (4)	136

Symmetry codes: (i) −*x*, −*y*, −*z*+1; (ii) −*x*, −*y*+1, −*z*+1.
